# Evaluation of Plant-Derived Promoters for Constitutive and Tissue-Specific Gene Expression in Potato

**DOI:** 10.3390/plants9111520

**Published:** 2020-11-09

**Authors:** Dmitry Miroshnichenko, Aleksey Firsov, Vadim Timerbaev, Oleg Kozlov, Anna Klementyeva, Lyubov Shaloiko, Sergey Dolgov

**Affiliations:** 1 Branch of Shemyakin and Ovchinnikov Institute of Bioorganic Chemistry RAS, 142290 Pushchino, Russia; aleksey_firsov@mail.ru (A.F.); timerbaev@gmail.com (V.T.); oleg632@ya.ru (O.K.); anutik.vlasowa@yandex.ru (A.K.); shaloiko@yandex.ru (L.S.); dolgov@bibch.ru (S.D.); 2 All-Russia Research Institute of Agricultural Biotechnology, Timiryazevskaya Street 42, 127550 Moscow, Russia

**Keywords:** *Solanum tuberosum*, genetic transformation, promoter choice, organ-specific

## Abstract

Various plant-derived promoters can be used to regulate ectopic gene expression in potato. In the present study, four promoters derived from the potato genome have been characterized by the expression of identical cassettes carrying the fusion with the reporter β-glucuronidase (*gusA*) gene. The strengths of *StUbi*, *StGBSS*, *StPat*, and *StLhca3* promoters were compared with the conventional constitutive CaMV *35S* promoter in various organs (leaves, stems, roots, and tubers) of greenhouse-grown plants. The final amount of gene product was determined at the post-transcriptional level using histochemical analysis, fluorometric measurements, and Western blot analysis. The promoter strength comparison demonstrated that the *StUbi* promoter generally provided a higher level of constitutive β-glucuronidase accumulation than the viral CaMV *35S* promoter. Although the *StLhca3* promoter was predominantly expressed in a green tissue-specific manner (leaves and stems) while *StGBSS* and *StPat* mainly provided tuber-specific activity, a “promoter leakage” was also found. However, the degree of unspecific activity depended on the particular transgenic line and tissue. According to fluorometric data, the functional activity of promoters in leaves could be arranged as follows: *StLhca3* > *StUbi* > CaMV *35S* > *StPat* > *StGBSS* (from highest to lowest). In tubers, the higher expression was detected in transgenic plants expressing *StPat-gusA* fusion construct, and the strength order was as follows: *StPat* > *StGBSS* > *StUbi* > CaMV *35S* > *StLhca3*. The observed differences between expression patterns are discussed considering the benefits and limitations for the usage of each promoter to regulate the expression of genes in a particular potato tissue.

## 1. Introduction

The potato is an essential vegetable crop in various climate areas. The global production of potatoes takes first place among dicotyledonous species, second only to monocotyledonous cereal crops such as wheat, rice, and corn [1]. It is generally consumed in fresh, frozen, and dehydrated food products and food ingredients but is also used as animal feed and for industrial purposes as a source of starch, bio-ethanol, and other nutrients. To meet various demands, potato breeders are focused on modifying diverse traits ranging from the improved nutritional content of tubers to pathogen, insect, and viral resistance and overall plant performance under stress.

In recent decades, the advanced methods of genetic modifications have become increasingly important in modern breeding programs. Such technologies as transgenesis and genome editing provide the specific alteration of potato genome that is not easily reached using conventional breeding methods [2]. Many factors influence the success of these powerful technologies in potatoes. Modification of gene function is generally achieved by the temporal or stable expression of genetic vectors. The coding sequences of vectors are driven by promoters to control expression in plant cells. It is important to target the expression of genetic sequences to particular tissues or organs during specific developmental stages of the plant and to avoid possible undesirable effects. The choice of an appropriate promoter is therefore an important element in the accurate modification of gene function in engineered plants. 

To date, the majority of transgenic potato plants have been produced using the constitutive *35S* promoter of *Cauliflower Mosaic Virus* (CaMV) as a main genetic element for regulating transgene expression. Although the CaMV *35S* promoter remains the most popular in potato and other dicotyledonous species, the native promoters have become more in demand to control the temporal and spatial efficiency of expression [3,4,5,6]. The replacement of viral or foreign promoters by promoters derived from the same species allows the production of cisgenic or intragenic plants, which are free from unwanted foreign sequences [7]. This, in turn, can simplify the regulation and commercialization of genome-engineered crops. The use of a range of plant-derived promoters also contributes to the successful multi-gene transformation, which is aimed at the production of plants with stacked traits or at engineering metabolic pathways by simultaneous introduction and expression of several genes. It is highly recommended to drive introduced sequences under different promoters in order to avoid accidental gene silencing due to the homology of repetitious promoters [3,8,9]. 

Various plant-derived promoters have already been validated to provide different transgene expression patterns in potato. The number of promoters derived from the potato genome is still limited, but a few of them were successfully applied for genetic engineering. The best-known potato promoters are promoters that drive tuber-specific expression. To date, several promoters have been characterized, including promoters of such genes as patatin [10], granule-bound starch synthase I (GBSSI) [11], laccase [12], and glucose pyrophosphorylase [13]. Both patatin and GBSS promoters have already been successfully used for metabolic engendering in potato to enhance the tuber content of oil [14,15], carotenoid [16], amylose [17] and cyanophycin [18] and for the production of edible vaccines [19]. Under tuber-specific transcriptional control of potato glucose pyrophosphorylase promoter, a reduction in the acrylamide content in fried potatoes was also achieved [13]. 

The promoters expressed in green tissues also play an important role in potato transgenesis. Green tissues, especially foliage, are the main target for viral, pathogenic, and insecticidal attacks in potato. Generally, the viral CaMV *35S* promoter is used to achieve the overexpression or knockout of genes involved in the resistance of potato to biotic stresses. In a few cases, the promoters derived from the housekeeping genes, such as ubiquitins, have been characterized and used as alternatives to the CaMV *35S* promoter [20,21,22]. The use of constitutive promoters was beneficial when the expressed sequences did not affect the function of the potato’s own genes. In applications targeted to regulate certain key gene(s), such as transcription factors or metabolic genes, the constitutive tissue-independent expression under the CaMV *35S* promoter caused some imbalance in transgenic plants, negatively affecting physiological processes and tuber production [10,23]. Unwanted effects may be prevented or lowered by the application of promoters targeted on the expression in foliage, avoiding expression in the edible tubers. For example, a light-inducible foliage-specific promoter of potato *Lhca3* gene (chlorophyll a/b binding protein; apoprotein II of the light-harvesting complex of photosystem I) was identified and included in expression cassettes to control tuber moth attacks [24] and blackleg disease [25] and to limit viral infection [26]. Alternatively, other environmentally inducible promoters with different levels of activity might be adopted to minimize the adverse effects. However, the practical use of inducible promoters is strictly limited, since only a few of them, such as cold- or pathogen-inducible promoters, have been isolated from the potato genome [27,28]. 

The aim of this work was to test and compare the efficacy of several potato-derived promoters with an aim to apply them in various functional and applied studies. It should be noted that in most published reports, the potato promoters were readily compared with the CaMV *35S* promoter, while the direct comparison between the promoters is rarely performed. Additionally, it is rather problematic to compare particular reports as varied expression cassettes, potato varieties, and environmental conditions were used to characterize the activity and strength of promoters. 

In our study, we simultaneously analyzed the activities of four promoters isolated from the potato genome. These include two tuber-specific constitutive promoters (*StPat* and *StGBSS*) and two promoters targeted for expression in foliage, such as the constitutive *StUbi* promoter and the light-inducible *StLhca3* promoter. The activity of promoters was determined at a post-transcriptional level using promoter–*gusA* fusion and compared with the identical CaMV *35S*–*gusA* construct. 

## 2. Materials and Methods 

### 2.1. Plasmid Construction

The promoter regions of the *StLhca3* gene (chlorophyll a/b binding protein; apoprotein II of the light-harvesting complex of photosystem I, GenBank no. S66876) and the *StUbi* gene (polyubiquitin gene, GenBank no. U26831) were amplified from genomic DNA of potato cv. “Manhattan”. Promoters of tuber-specific class-I patatin gene (*StPat*) (GenBank no. A08215) and granule-bound starch synthase (*StGBSS*) genes (GenBank no. A23740) were cloned from genomic DNA of potato cv. “Chicago”. The proof-reading Phusion high-fidelity DNA polymerase (Thermo Fisher Scientific, Waltham, USA) was used to amplify the promoter sequences by using PCR primers as detailed in Table S1. Produced fragments were cloned into the intermediate vector pUC18 and sequenced to ensure fidelity. 

**Figure 1:**
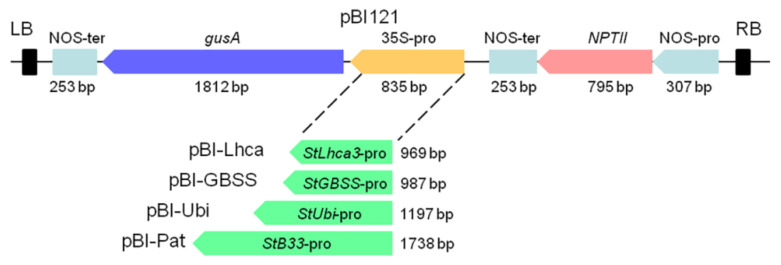
Schematic representation of expression cassettes carrying the potato promoter–*gusA* fusions. Binary vector pBI121 was used as the base vector. *StLhca3, StGBSS*, *StUbi*, and *StPat* promoters were inserted into pBI121 in the place of the CaMV *35S* promoter to generate plasmids pBI-Lhca, pBI-GBSS, pBI-Ubi, and pBI-Pat, respectively.

A modified binary construct pBI121 [29] in which the CaMV *35S* promoter driving the *gusA* gene was removed was used to create constructs for transformation. Promoter fragments of the *StGBSS*, *StPat*, *StLhca3*, and *StUbi* were released from the relevant intermediate vectors and then placed upstream of the *gusA* reporter gene with the Hind*III*—Xba*I* (*StGBSS*) or *Hind*III—*BamH*I (*StPat*, *StLhca3*, *StUbi*) sites. The resulting constructs were designated as pBI-Pat, pBI-GBSS, pBI-Ubi, and pBI-Lhca (Figure 1) and were transformed into *Agrobacterium tumefaciens* strain AGL0.

### 2.2. Production of Transgenic Plants

Potato (*Solanum tuberosum*) cv. “Chicago” provided by the Doka-Gene Technology Ltd. (Rogachevo, Russia) was used for genetic transformation. Plants were maintained in vitro in plastic containers under controlled conditions (16 h light 40 µmol m^−2^ s^−1^ and 8 h dark at 22–25 °C) on phytohormone-free Murashige and Skoog (MS) basal medium supplemented with 3% sucrose and 7% agar, pH 5.8. Stem internodal segments (0.5–1 cm long) of 3–4-week-old micropropagated plants were used as explants for transformation. The inoculation and cocultivation of explants with *Agrobacterium* were performed as described previously [23]. Shoot regeneration and transgenic plant selection were carried out under the light (16 h light 40 µmol m^−2^ s^−1^ and 8 h dark at 22–25 °C) on the MS-basal medium supplemented with zeatin-riboside (3 mg L^−1^), GA_3_ (1.0 mg L^−1^), indoleacetic acid (2 mg L^−1^), kanamycin (50 mg L^−1^), and cefotaxime (500 mg L^−1^). Every 12–15 nodal explants were subcultured on fresh regeneration medium in Petri dishes every 10 days. After the third subculture, the selective concentration of kanamycin was increased to 75 mg L^−1^. Only one kanamycin-resistant shoot was collected from each explant to avoid duplication of transgenic lines. Independent shoots were multiplicated in culture vessels on phytohormone-free MS medium supplemented with 150 mg L^−1^ cefotaxime and 75 mg L^−1^ kanamycin. 

Regenerated shoots, which were capable of developing good root systems on the selective medium, were checked by PCR for the insertion of promoter–*gusA* fusion sequence. To identify the events, the primer corresponding to the sequence from *gusA* gene was used as a reverse primer, while the sequences corresponding to the promoter regions were used as a forward primer (see Table S1). 

### 2.3. Histochemical and Fluorometric β-glucuronidase (GUS) Assays

The staining of various tissues of transgenic potato was carried out according to [29]. Histochemical staining was performed with 5-bromo-4-chloro-3-indolyl-D-glucuronide (XGluc, Sigma-Aldrich, St. Louis, USA) as the substrate. After staining, the samples were subsequently cleared through an 80% ethanol series at 4 °C. Fluorometric assays were performed as described [29] with the following modifications. Leaves and tubers of the greenhouse-grown transgenic and non-transformed plants were used for protein extraction. The tissue samples (1 g) were powdered in liquid nitrogen, and 100 mg of ground material was resuspended in four volumes of GUS extraction buffer containing 50 mM Na_3_PO_4_ (pH 7.0), 10 mM β-Mercaptoethanol, 10 mM Na_2_EDTA, 0.1% Sodium lauryl sarcosine (*v/v*), 0.1% Triton X-100 (*v/v*). Total protein was extracted for 45 min at 4 °C. After centrifugation for 20 min at 16,000 g at 4 °C, the supernatant was immediately used for analysis. Protein concentration was measured by DC protein assay kit (BioRad, Hercules, USA). Four-methyl umbelliferyl β-D-glucuronide (Sigma-Aldrich, St. Louis, USA) was used in the fluorometric assay; to generate the fluorescence calibrate curve, 4-methyl-umbelliferone (Sigma-Aldrich, St. Louis, USA) was used as standard. GUS activity was quantitatively determined using fluorometer FLUOstar OPTIMA (BMG Labtech, Ortenberg, Germany) and calculated as nmol of 4-methyl-umbelliferone (4-MU) produced per sec per µg of total soluble protein (TSP), noted as units thereafter. For each promoter construct, ten independent transgenic lines were analyzed. Three individual plants of each line were used as biological replicates.

### 2.4. Western Blot Analysis 

Total soluble protein (TSP) was extracted as described above for fluorometric assays. A protein sample (25 µg) from each studied line was separated by 12% SDS-PAGE and transferred onto an NC membrane (BioRad, Hercules, USA). Rabbit anti-β-glucuronidase (diluted 1:2000, Sigma-Aldrich, St. Louis, USA) polyclonal antibodies served as the primary antibodies. Anti-rabbit IgG conjugated to alkaline phosphatase was used as the secondary antibody (1:4000, Pierce, Waltham, USA). Blots were treated with nitroblue tetrazolium and 5-bromo-4-chloro-3-indolyl phosphate (BCIP) for visualization.

## 3. Results

### 3.1. Generation of Transgenic Potato Plants 

**Figure 2:**
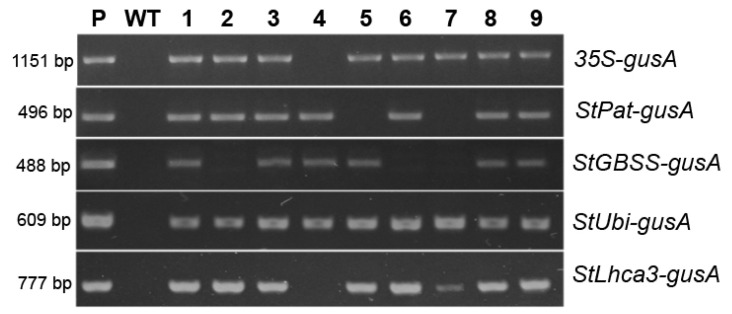
*nptII*-positive transgenic potato plants produced after Agrobacterium-mediated transformation were analyzed for the presence of the promoter–*gusA* fusion by PCR amplification; an example of analysis for a part of independent lines (labeled as 1–9); lane P, the plasmid DNA of the corresponding vector; lane WT, untransformed potato plant.

The five vectors, outlined in Figure 1, were transformed into potato cultivar “Chicago”, and transgenic plants were generated on the medium with a lethal dose of kanamycin, resulting in 27 to 71 independent antibiotic-resistant plants being obtained for each construct within three months of selection (Table 1). The initial PCR screening of the regenerated plantlets confirmed that all analyzed plants were transgenic, as they contained the inserts of selective *nptII* gene (data not shown). The efficiency of *Agrobacterium*-mediated transformation was in the range of 69–77% for pBI121, pBI-Pat, pBI-Ubi, and pBI-Lhca constructs, while the pBI-GBSS vector displayed weak transformation efficiency of 25% (Table 1). Moreover, a significant part of the *nptII*-positive pBI-GBSS plants carried the incorrect insertion of the sequence of promoter–*gusA* fusion (Figure 2). Likewise, most of the plants obtained using the pBI-Pat vector had truncated inserts, and only a quarter of the *nptII*-positive plants were identified as containing the entire *StPat* promoter–*gusA* fusion inserts. At the same time, the correct insertions of promoter–*gusA* cassette were detected in the majority of kanamycin-positive transgenic plants generated after transformation with pBI121, pBI-Ubi, and pBI-Lhca vectors (Figure 2). Further analysis showed that only positive plants carrying the correct promoter–*gusA* inserts were involved. For each construct, ten independent lines were randomly selected among the transgenic plants and for their promoter activity.

### 3.2. GusA Expression Patterns in Various Tissues and Organs of Transgenic Potato Plants 

**Figure 3:**
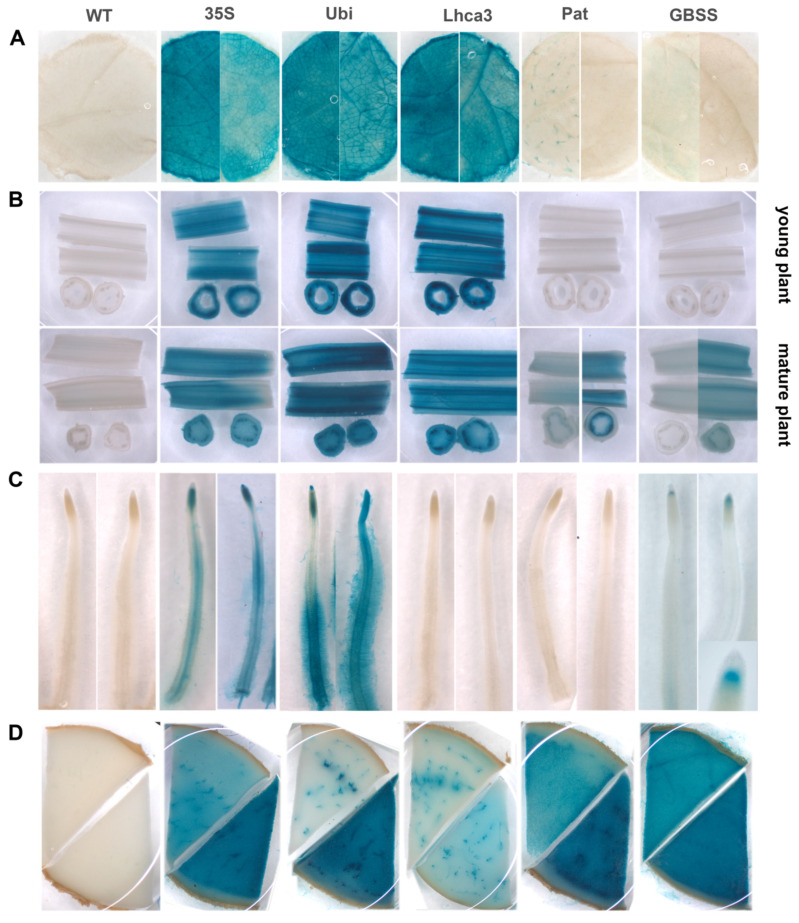
Expression pattern of *gusA* gene fused with the CaMV *35S*, *StUbi*, *StLhca3*, *StPat*, and *StGBSS* promoters in various tissues of transgenic potato plants and untransformed potato plant (WT), including leaves (**A**), young and mature stems (**B**), roots (**C**), and tubers (**D**). The characteristic tissue samples are shown; distinctive variations of GUS activity, if observed, are presented for two samples taken from independent lines.

Transgenic potato lines containing different promoter–*gusA* constructs were histochemically analyzed for organ- and tissue-specific GUS activity in leaves, young and old stems, tubers, and roots (Figure 3). In general, the activities observed in leaves of potato lines transformed with pBI-Ubi and pBI-Lhca constructs were higher compared to the CaMV *35S* promoter, although the spatial pattern of expression was comparable (Figure 3A). The lines showed the most intense staining in veins, midribs, and ribs and less intense staining of lamina tissues. In contrast, visible expression of the *gusA* gene under the control of *StGBSS* and *StPat* promoters was very weak in leaves and observed in a few independent lines. GUS staining was barely noticeable in mesophyll tissues and some trichomes but was never seen in vascular tissues (Figure 3A).

Longitudinal and cross-sections of stems of transgenic lines carrying the fusion with *StUbi*, *StLhca3*, and CaMV *35S* promoters revealed blue staining in all cells analyzed; however, the strongest expression was observed in the vascular tissue (Figure 3B). Generally, the CaMV *35S* promoter was expressed less strongly, while the *StLhca3* and *StUbi* promoters displayed equally strong activity in young and old tissues. GUS staining was not observed in young stems of transgenic plants transformed with *Pat* and *GBSS* promoter–*gusA* constructs; several transgenic lines, however, later displayed the GUS activity in the mature stems (Figure 3B). The degree of *gusA* expression in these lines was not as strong as in old stems of lines transformed with pBI-Ubi, pBI121, and pBI-Lhca constructs, and the blue staining was predominately accumulated in phloem bundles and bundle sheath cells (Figure 3B).

The analyzed promoters showed distinct localization of *gusA* expression in the roots (Figure 3C). No histochemical staining for GUS activity was observed in transgenic lines containing pBI-Lhca and pBI-Pat constructs. In case of constitutive CaMV *35S* and *StUbi* promoters, intensive blue staining was easily observed in various tissues and parts of roots, including dividing cells of distal and proximal meristems, columella root cap, steel and pericycle cells in the elongation and differentiation zone, as well as in root hairs cells (especially in transgenic lines with pBI-Ubi construct). Surprisingly, the *StGBSS* promoter strictly directed the *gusA* gene expression to the “stem cell niche” (the quiescent center and adjacent initials) of root apical meristem, while the other developing and mature root tissues were lacking GUS activity (Figure 3C).

All the analyzed promoters showed GUS enzymatic activities in tubers; however, the intensity of staining significantly differed among the promoters and between independent transgenic lines transformed with the same construct. As shown in the characteristic transverse sections through the middle part of the potato tubers (Figure 3D), GUS activity under the control of *StLhca3* and *StUbi* promoters was more specific to the vascular bundles and surrounding cells, while the storage cells of the perimedullary zone and pith showed much less intensity of staining. Transgenic tubers of plants transformed with *StPat* and CaMV *35S* promoters demonstrated a similar spatial pattern of expression, with the exception that the GUS accumulation in storage parenchyma cells of the tuber core was much higher. In contract, transgenics with the *StGBSS* promoter showed high GUS activity without a pronounced difference between the vascular and storage tissues. In the case of CaMV *35S*, *StGBSS* and *StPat* promoters, the cells of the primary cortex demonstrated lower GUS accumulation than other tissues of the tuber; therefore, the intense blue staining did not spread beyond the vascular ring even in highly expressing transgenic lines (Figure 3D).

### 3.3. Quantitative Analysis of Promoter–gusA Activity in Transgenic Potato Plants

**Figure 4:**
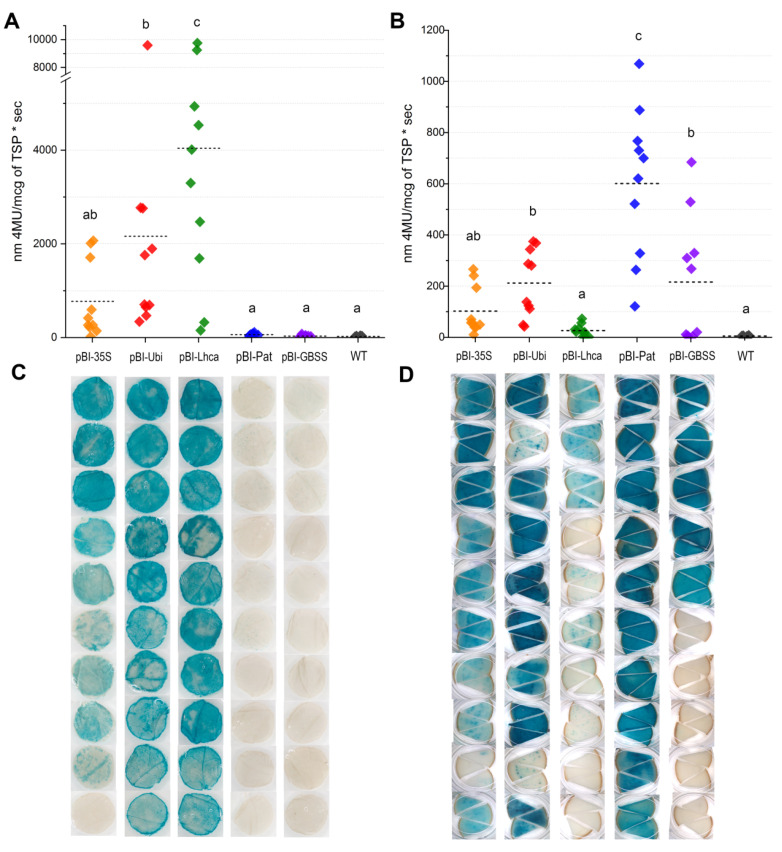
GUS activity expressed using various promoters in leaves (**A**,**C**) and tubers (**B**,**D**). GUS activity is measured fluorometrically (**A**,**B**) as nm 4-MU/mcg of TSP • sec; rhombuses indicate the value of GUS activity for one independent line measured in 3 individual plants per line; horizontal bars represent the average GUS activity for the promoter; for each promoter, ten independent lines carrying the correct inserts of a promoter–*gusA* fusion were analyzed. The pattern of histochemical GUS accumulation (**C**,**D**) is shown for each independent transgenic line; the characteristic tissues samples are presented; samples in the same row of panels c and d belong to the same transgenic line of the analyzed promoter.

Quantitative GUS enzymatic assays were performed on leaves and tubers of the ten transgenic greenhouse-grown plants that were previously analyzed for GUS staining. Considering the differences in GUS activity between the transgenic potato lines, the data are presented in terms of mean values for each promoter construct (Table 2, Figure 4A,B). The fluorometric data were generally consistent with GUS histochemical staining; however, the values varied greatly depending on the line and promoter.

According to the measurements, the higher GUS activity in leaves was directed by the pBI-Lhca construct and yielded an average of 4045.2 units (4-MU/mcg of TSP sec), while the values between the individual independent plants could differ more than 60 times (Table 2, Figure 4A). In the case of the constitutive CaMV *35S* promoter, the values of *gusA* expression were in the range of 141.9–2068.5 units, and overall activity yields were only 771.9 units. The plants with *StUbi* promoter–*gusA* construct had two-fold lower average activity in leaves (2163.6 units) than the plants expressing *StLhca3* promoter–*gusA* cassette, although the difference between the highest active transgenic lines was marginal (9752.3 units for *StLhca3* promoter and 9592.4 for *StUbi* promoter) (Figure 4A).

In contrast, tuber-specific promoters displayed extremely low fluorometric GUS activity (Figure 4A,C), which ranged from 11.8 to 75.0 units and from 28.4 to 110.5 units in leaf extracts of the lines with *StGBSS* and with *StPat* promoters, respectively. Due to low fluorometric values, the difference from the untransformed potato (WT) was not statistically significant for the majority of the lines (15 lines) transformed with the pBI-Pat and pBI-GBSS cassettes. Only a few independent lines with residual blue GUS staining in leaf tissues showed significantly higher GUS activity than WT; however, the fluorimetric values were lower than those observed for protein extracts of lines with the weakest *gusA* expression driven by the CaMV *35S*, *StUbi*, and *StLhca3* promoters (Table 2).

In tubers, the strength of *StGBSS* and *StPat* promoters increased significantly, and the fluorometric analysis was consistent with histochemical observation for both promoters (Figure 4B,D). All the studied lines carrying *StPat* promoter–*gusA* cassette demonstrated good accumulation of GUS; in contrast, half of the lines with *StGBSS* promoter–*gusA* sequence showed very low or no detectable activity. The quantitative data showed that among studied promoters, the *StPat*-driven expression of the *gusA* gene was the highest; on average, 598.8 units per line were detected with a maximum of 1068.4 units in one of the lines. The *StLhca3* promoter drove the lowest GUS activity in tubers; the average value was only 26.0 units, with a maximum of 72.7 units in the most expressing line. The ability of constitutive promoters to drive *gusA* expression in tubers was more stable compared to the *StGBSS* promoter, since almost all of the analyzed lines displayed obvious GUS activity (Figure 4B,D). 

In the case of CaMV *35S* promoter–*gusA* fusion, the levels of GUS accumulation in transgenic tubers ranged from 10.6 to 266.3 units, with an average value of 102.4 units. *StUbi* promoter drove somewhat higher *gusA* expression; on average, 211.2 units per analyzed line were detected, with the means ranging from 42 to 375 units in individual lines. Although the fact that the blue staining in tuber tissues of the most expressing *StUbi* promoter–*gusA* lines seemed more intensive than in the lines demonstrating the activity of *StGBSS* promoter–*gusA* cassette, the fluorometric data were comparable or even higher for the *StGBSS* promoter, since the values varied within 267.8–684.1 units. Therefore, excluding the silencing lines, the average level of GUS accumulation in active *StGBSS* promoter–*gusA* lines (n = 5) was 364.2 units (Table 2), which is approximately 1.75 times higher than in the lines expressing pBI-Ubi construct (n = 10), but 1.6 times lower compared with the plants expressing the pBI-Pat construct (n = 10). 

In general, the expression driven by *StGBSS* or *StPat* promoters was enhanced in tubers, while CaMV *35S*, *StUbi*, and *StLhca3* promoters directed higher GUS accumulation to leaves than to tubers. In the case of the CaMV *35S* promoter, there was a trend for conformity of GUS activity levels in various tissues of transgenic lines. The three most expressing lines showed equally high *gusA* expression in both leaves and tubers, whereas lines with lower GUS accumulation in leaves showed lower activity in tubers. In contrast, the higher expression in leaves of some lines carrying the *StUbi* promoter–*gusA* or *StLhca3* promoter–*gusA* fusions did not always correspond to a higher GUS accumulation in tubers.

**Figure 5:**
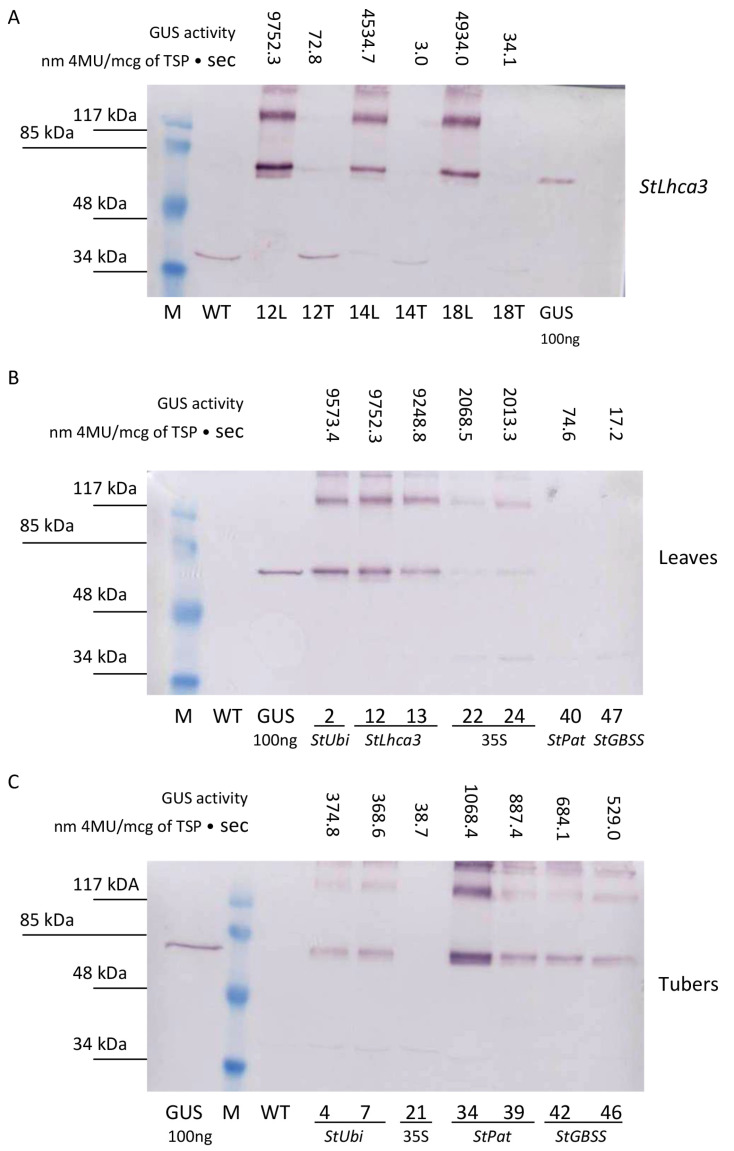
Quantitative comparison of promoter–*gusA* activities in transgenic tissues of the independent potato lines. An example of the Western blot analysis for the presence of GUS in total protein fractions extracted from leaves (L) and tubers (T) of the same transgenic lines carrying the *StLhca3* promoter–*gusA* fusion (**A**). Comparative levels of GUS accumulation in protein extracts are taken from the various transgenic lines characterized by different levels of fluorometric GUS activity in leaves (**B**) and tubers (**C**).

For a more detailed study of GUS accumulation, a comparative Western blot analysis was performed on protein extracts taken from the lines characterized by different levels of fluorometric GUS activity (Figure 5). The accumulation of GUS protein was clearly detected in the samples of total protein fractions extracted from leaves of transgenic lines carrying the *gusA* gene driven under the control of the *StLhca3*, *StUbi*, and CaMV *35S* promoters (Figure 5A,B). As expected, no bands were recognized in leaf extracts of the lines carrying the inserts of the *gusA* fusion with the tuber-specific promoters *StPat* and *StGBSS*, as well as in the protein samples from wild-type potato. In general, the data from the Western blot analysis correlated with the fluorometric data. For example, a semi-quantitative comparison of the selected *StLhca3-gusA* lines showed that the intensity of bands was strongest for the leaves extracts, while it was hard to find the specific protein band in the tuber extracts (Figure 5A).

When the protein fractions were extracted from tubers, the bands corresponding to GUS protein were identified in lines generated after transformation with pBI-Ubi, pBI-Pat, and pBI-GBSS vectors that showed a higher tuber-specific promoter activity (Figure 5C). The intensity of bands was strongest for lines transformed with *StPat* promoter–*gusA* fusion, followed by lines transformed with *StGBSS* promoter–*gusA* and *StUbi* promoter–*gusA* constructs (Figure 5C). In contrast, it was difficult to detect the accumulation of GUS protein in tuber extracts of potato lines transformed with *StLhca3* promoter–*gusA* construct (Figure 5A) and lines with low tuber-specific activities (Figure 5C). As in the case of leaf extracts, the intensity of the bands generally correlated with the fluorometric data.

## 4. Discussion

Modern strategies for the breeding of potato varieties rely on the successful manipulation of the potato’s own or foreign genes [2,30]. Effective expression systems should be designed to increase the productivity of potato plants and alter their metabolic activity for both food-based agricultural and non-food industrial purposes. The appropriate promoters are crucial to providing the desired pattern of expression, so their detailed characterization could help to properly manipulate the functional activity of genes of interest. 

Owing to the increased demand for exploiting potato tubers as a production platform for nutrients with pharmaceutical or industrial interest, the choice of controllable promoter displaying tuber-specific expression is important for the creation of “factory” plants. Though both *StBGSS* and *StPat* promoters are known to be suitable for such purposes [14,15,16,17,18,19], in our study, the tuber-specific activity of *StPat* promoter (1738 bp) was generally higher and more stable than the activity of *StGBSS* promoter (936 bp). Patatin is one of the main storage proteins in potato tubers, and 98–99% of patatin transcripts are accumulated in tubers. In contrast to patatin genes, the expression of granule-bound starch synthase (*GBSS*) genes is not strictly associated with tuberization. Since the activity of *GBSS* genes is related to amylose biosynthesis during starch granulation, genes in the *GBSS* family are known to be expressed in various storage tissues, especially in leguminous seeds and cereal grains [31]. The promoters of class-I patatin genes are characterized by the presence of a range of *cis*-regulatory sequence motives, such as K, M, and D boxes and tandemly arranged sequences [32,33,34]. It was confirmed that *cis*-elements responsible for the tuber-specific and sucrose-responsive activity of class-I patatin genes are located between the nucleotides –252 and +14 (relative to the transcription initiation site), thus the deletion of this fragment significantly decreased the expression level [14,31,35]. On the other hand, the levels of GUS activity in different potato tissues were found to be significantly higher for the longer patatin promoter (2225 bp) than for the shorter one (1215 bp) [35], indicating that the presence of additional *cis*-regulatory elements located in the further upstream region may positively influence the strength of activity. To drive the tuber-specific expression of potato *GBSSI* genes, the 0.4 kb region (–346 bp until +54 bp) of the promoter sequence was found to be required [36]; in cereals, however, the *cis*-acting elements, responsible for the grain-specific *gbss1* transcription, are located −1.9 kb upstream of the promoter [37]. In our study, the 936 bp variant of the *StGBSS* promoter successfully controlled tuber-specific accumulation of the *gusA* protein with rather low non-tuber-specific activities; its strength, however, was lower than the activity of the 1738 bp *StPat* promoter. The *StPat* promoter also showed a higher level of accumulation in other tissues. 

In theory, the ideal tuber-specific promoter should not display functional activity in other tissues. In the present study, both *StPat* and *StGBSS* promoters contributed to the leakiness of GUS activity. The unspecific activity was primarily found in old stem tissues, and as we indicated earlier, *StGBSS* promoter was less active, especially in the young stem tissues. These data are in agreement with those of Visser et al. [38], who previously reported that the bottom part of stems (which are supposed to be older tissues) accumulated a higher amount of protein than the top parts (younger tissues) when the *StGBSS* promoter was used to drive the *gusA* gene. In contrast, Bansal et al. did not observe *StGBSS*-driven *gusA* expression in stems, or in roots and leaves, at least upon histochemical analysis [11]. In their study, however, the number of transgenic plants was restricted. In our study, most of the independent lines also showed no functional activity in leaves; however, several lines carrying *StGBSS* promoter–*gusA* cassette still displayed a low GUS accumulation. At the same time, the root-specific activity of *StGBSS* promoter was much lower than reported earlier [38], and it was found only in a very specific area of young roots. Patatin promoters were also reported to exhibit some transcriptional activity in roots [35,39], stolons [35,39], and leaves [10]. Aminedi and Das previously reported that a leaking pattern in stolons was more evident for the shorter (360 bp) version of the patatin promoter, while the longer versions (674–3500 bp) demonstrated a lower level of “leakiness”, with higher tuber-specific activity [39]. Using the 1738 bp version of the *StPat* promoter, we observed the accumulation of GUS protein in stems and a certain activity was also detected in leaves, but we could not find any histochemical activity of β-glucuronidase in roots. The different genotypes, structural variation in promoters and expression cassettes used in earlier reports, and a sometimes insufficient number of analyzed transgenic lines could explain the contradictory results.

In the present study, the construct with the *StGBSS* promoter has a clear tendency for silencing as half of the analyzed lines displayed no functional activity of a driven gene. Previously it was shown that different regions of the *StGBSS* promoter used in RNAi cassettes may influence the level of internal silencing of the *StGBSS* gene, and the various sequence motives may induce silencing effects in up to 57–60% of produced plants [40]. The application of the *StGBSS-*promoter-based construct in the present study significantly affected the overall output of independent transgenic events as well, since the transformation efficiency with pBI-GBSS vector was considerably lower compared to the other constructs. For the *StPat*-based construct, the difficulties with the production of functionally active transgenic plants were also found. After the transformation with pBI-Pat vector, a significant part of generated potato plants had truncated variants of *StPat* promoter–gusA sequence, so a higher number of independent lines was required to identify the events with the correct insertion of *StPat* promoter–*gusA* fusion. Interestingly, such insertional and transformation problems were not observed for other constructs, indicating that some additional aspects are present, which are associated with the natural homology between the endogenic allelic sequences of tuber-specific promoters.

Compared to the tuber-specific promoters, the inclusion of the *StUbi* or *StLhca3* potato promoters into the transformation vector affected neither the transformation frequency nor the functional activity of transgenic lines. Previously, the promoter of the *Lhca3*.St.1 gene was shown to predominantly express in foliage and stems and was not expressed in tubers or roots of potato [41]. Our results are partially consistent with these observations. No activity was detected in roots of transgenic lines expressing the *StLhca3* promoter–*gusA* construct, though the evident “leakage” phenomenon was discovered in the tubers (Figure 4C). The nonspecific accumulation of GUS in tubers was 100–180 times lower than in leaves and was found mainly in vascular bundles of tubers, but in general, this contradicts previous observations [25,41]. It is hard to speculate whether unspecific GUS accumulation in our study was the result of the vascular transport from the “green” parts, as the tubers subjected to analysis were stored in the dark. Further research is required to clarify the regulatory mechanisms controlling the unspecific activity of the *StLhca3* promoter in potato tubers.

Our findings demonstrated that the *StLhca3* promoter is the best candidate to achieve robust expression in potato leaves. There were no comparative data for its functional activity in potato; however, it has been reported to provide much better *gusA* expression than CaMV *35S* promoter in chrysanthemum [42] and tobacco [41]. The data from the present study support this tendency, as the constitutive expression of *StUbi* and CaMV *35S* constructs in potato leaves did not exceed that of the *StLhca3* promoter–*gusA* fusion. This difference in expression levels is likely associated with a better stability of the mRNAs, due to potential post-transcriptional modifications attributed to *StLhca3* promoter [3].

The comparison between constitutive promoters indicates an advantage of the *StUbi* promoter over the viral CaMV 35S promoter for providing a robust expression in potato tissues. In our study, transgenic potato plants expressing *StUbi* promoter–*gusA* construct displayed 1.5–10 times higher GUS activity than the CaMV 35S promoter in both aerial and underground tissues. Such comparison has not been previously carried out on potato. The results presented here are generally in agreement with comparable studies performed on various species involved CaMV *35S* promoter and plant-derived promoters of polyubiquitin genes [3,6,8,9,43]. According to the presented data, the constitutive promoters provided a higher level of *gusA* expression in leaves than in the tubers of potato. Surprisingly, tubers generally accumulated less GUS protein than leaves, even in the highly productive plants expressing tuber-specific *StPat* promoter–*gusA* and *StGBSS* promoter–*gusA* constructs. This observation, however, contradicts some studies reporting that the accumulation of protein in tubers provided by the tuber-specific promoters was higher than or equal to GUS accumulation mediated by constitutive promoters in leaves [38,39]. An important aspect to consider is that in addition to tuber-specific *cis*-elements, the promoters of *Patatin* and *GBSS* genes also include the sucrose-inducible specific motifs; therefore, increased the expression has been achieved mainly in micro-tubers of potato produced by cultivating plants in vitro under the influence of higher concentrations of sucrose [34,39].

Though the CaMV 35S promoter is commonly used in potato genetic transformation to drive expression throughout the potato plant, in the present study, it demonstrated a moderate expression ability both in leaves, as compared to the *StLhca3* and *StUbi* promoters, and in tubers, as compared to the *StPat* or the *StGBSS* promoters. Thus, it seems appropriate to use the stronger organ-specific promoters, such as *StLhca3* promoter to maximize the expression of heterologous genes in leaves or the *StPat* and the *StGBSS* promoters to regulate expression in tubers. Our recent results confirm that “green tissue”-specific properties of *StLhca3* promoter may provide benefits in genetically engineering plants to protect potato from foliar-associated diseases [26].

The results obtained here demonstrate a considerable variation in *gusA* expression between the independently generated transgenic lines from a very high level of protein accumulation down to its absence. We associate this variation with the known “position effect”, in which the expression of a heterologous gene depends on the place of its integration into the plant genome. In our study, the analysis of 10 independently produced transgenic lines made it possible to identify 1–3 lines with a high expression for each promoter variant. This indicates that the examination of a certain number of transgenic potato lines is necessary both for correct interpretation of expression data and for the successful production of plants yielding a sustainable amount of heterologous proteins.

In summary, we compared the level of activity and the expression patterns of a popular viral CaMV *35S* promoter with four potato-genome-derived promoters. Various research groups have previously explored the functional activities of potato promoters; however, it is hard to find reports in which the different promoters are characterized in the same potato variety on physiologically equivalent tissues of mature plants under the same environmental conditions using identical expression cassettes. Based on the data presented here, we recommend replacing the CaMV 35S promoter with the *StUbi* promoter to ensure a higher constitutive expression in all vegetative tissues of potato, while the functional activity of *StLhca3* promoter could be used for more prominent “green tissue”-specific expression. The *StLhca3* promoter application, however, should be accompanied by careful selection among the independent transgenic events to ensure that expression does not occur in the edible tubers. In our study, the application of *StPat* and *StGBSS* promoters was not sufficient to provide strict tuber-specific patterns of expression in transgenic potato; nevertheless, fluorometric analysis of tuber extracts showed that the activity of the *StPat* promoter–*gusA* construct was more pronounced between the two promoters. Since both *StPat* and *StGBSS* displayed promoter leakage, it should be considered by the researchers in the case of metabolic engineering to avoid unwanted phenotypic effects. Some technical aspects revealed here, such as lower transformation rate (for the construct with the *StGBSS* promoter) and truncated insertions (for constructs with the *StPat* and *StGBSS* promoters), should be also taken into account. The results of this study provide important comparative information for designing constructs to produce transgenic and intragenic potato plants for basic research and commercial plant genetic engineering using promoters with a predicted tissue-specific expression.

## Figures and Tables

**Table 1:** Production of transgenic potato plants after Agrobacterium-mediated transformation.

Vector	Number of Explants	Kanamycin- Positive Shoots Regenerated	Number of Shoots			The Transformation Efficiency (%) **
			Analyzed by PCR	*nptII* Positive (%)	*gusA* Positive (%) *	
pBI121	51	36	15	15 (100)	14 (93)	71
pBI-Pat	92	71	43	43 (100)	12 (28)	77
pBI-GBSS	108	27	27	27 (100)	11 (41)	25
pBI-Ubi	76	57	18	18 (100)	18 (100)	75
pBI-Lhca	75	52	18	18 (100)	17 (94)	69

* Number of plants with a correct promoter–*gusA* insert. ** calculated as the percentage of the independent potato explants that produced *nptII* positive (PCR+) transgenic plants.

**Table 2:** GUS fluorescent quantitative analysis of the activity of *StUbi*, *StLhca3*, *StPat*, *StGBSS*, and CaMV *35S* promoters in transgenic potato plants grown under greenhouse conditions.

Promoter (Vector)	Tissue	Independent Lines	Average GUS Activities; Units *	Number of Lines Shown a Histochemical GUS Staining	Average GUS Activities Per Lines Showing a Histochemical GUS Staining; Units
WT **	Leaves	-	25.2 ± 3.5	0	-
	Tubers	-	4.8 ± 0.9	0	-
CaMV *35S* (pBI121)	Leaves	10	771.9 ± 259.0	9	855.3 ± 274.1
	Tubers	10	102.4 ± 29.6	10	102.4 ± 29.6
*StUbi* (pBI-Ubi)	Leaves	10	2163.6 ± 875.2	10	2163.6 ± 875.2
	Tubers	10	211.8 ± 41.9	10	211.8 ± 41.9
*StLhca3* (pBI-Lhca)	Leaves	10	4045.2 ± 1044.9	10	4045.2 ± 1044.9
	Tubers	10	26.0 ± 7.5	7	36.2 ± 7.8
*StPat* (pBI-Pat)	Leaves	10	63.4 ± 7.0	5	80.4 ± 8.3
	Tubers	10	598.8 ± 93.8	10	598.8 ± 93.8
*StGBSS* (pBI-GBSS)	Leaves	10	29.9 ± 5.9	3	51.5 ± 12.2
	Tubers	10	214.5 ± 78.6	5	364.2 ± 79.1

* Mean value of GUS activity ± SE was estimated in ten independent transgenic lines (3 individual plants/line, 10 lines for each construct), nm 4-MU/mcg of TSP • sec. ** Mean value of GUS activity ± SE in untransformed potato (WT) was estimated in five plants.

## Supplementary Materials

The following are available online at https://www.mdpi.com/2223-7747/9/11/1520/s1, Table S1: Primers used for molecular analysis of transgenic potato plants.

## References

[1] FAO http://www.fao.org/faostat/en/#data/QC/visualize.

[2] Hameed A., Zaidi S.S., Shakir S., Mansoor S. (2018). Applications of New Breeding Technologies for Potato Improvement. Front. Plant. Sci..

[3] Dutt M., Dhekney S., Soriano L., Kandel R., Grosser J.W. (2014). Temporal and spatial control of gene expression in horticultural crops. Hortic. Res..

[4] Ali S., Kim W.C. (2019). A Fruitful Decade Using Synthetic Promoters in the Improvement of Transgenic Plants. Front. Plant. Sci..

[5] Timerbaev V., Dolgov S. (2019). Functional characterization of a strong promoter of the early light-inducible protein gene from tomato. Planta.

[6] Kummari D., Palakolanu S.R., Kishor P.B.K., Bhatnagar-Mathur P., Singam P., Vadez V., Sharma K.K. (2020). An update and perspectives on the use of promoters in plant genetic engineering. J. Biosci..

[7] Holme I.B., Wendt T., Holm P.B. (2013). Intragenesis and cisgenesis as alternatives to transgenic crop development. Plant. Biotechnol. J..

[8] Peremarti A., Twyman R.M., Gómez-Galera S., Naqvi S., Farré G., Sabalza M., Miralpeix B., Dashevskaya S., Yuan D., Ramessar K. (2010). Promoter diversity in multigene transformation. Plant. Mol. Biol..

[9] Nuccio M.L., Lagrimini L. (2017). A Brief history of promoter development for use in transgenic maize applications. Maize. Methods in Molecular Biology.

[10] Liu Q., Guo Q., Akbar S., Zhi Y., El Tahchy A., Mitchell M., Li Z., Shrestha P., Vanhercke T., Liang G. (2017). Genetic enhancement of oil content in potato tuber (*Solanum tuberosum* L.) through an integrated metabolic engineering strategy. Plant. Biotechnol. J..

[11] Bansal A., Kumari V., Taneja D., Sayal R., Das N. (2012). Molecular cloning and characterization of granule-bound starch synthase I (GBSSI) alleles from potato and sequence analysis for detection of cis-regulatory motifs. Plant. Cell Tissue Organ. Cult..

[12] Ha J., Moon K., Kim M., Park S., Hahn K., Jeon J., Kim H. (2015). The laccase promoter of potato confers strong tuber-specific expression in transgenic plants. Plant. Cell Tissue Organ. Cult..

[13] Ye J., Shakya R., Shrestha P., Rommens C.M. (2010). Tuber-Specific Silencing of the Acid Invertase Gene Substantially Lowers the Acrylamide-Forming Potential of Potato. J. Agric. Food Chem..

[14] Liu X., Prat S., Willmitzer L., Frommer W. (1990). Cis regulatory elements directing tuber-specific and sucrose-inducible expression of a chimeric class I patatin promoter/GUS-gene fusion. Mol. Gen. Genet..

[15] Hofvander P., Ischebeck T., Turesson H., Kushwaha S.K., Feussner I., Carlsson A.S., Andersson M. (2016). Potato tuber expression of Arabidopsis WRINKLED1 increase triacylglycerol and membrane lipids while affecting central carbohydrate metabolism. Plant. Biotechnol. J..

[16] Diretto G., Al-Babili S., Tavazza R., Papacchioli V., Beyer P., Giuliano G. (2007). Metabolic Engineering of Potato Carotenoid Content through TuberSpecific Overexpression of a Bacterial Mini-Pathway. PLoS ONE.

[17] Andersson M., Melander M., Pojmark P., Larsson H., Bülow L., Hofvander P. (2006). Targeted gene suppression by RNA interference: An efficient method for production of high-amylose potato lines. J. Biotechnol..

[18] Hühns M., Neumann K., Hausmann T., Klemke F., Lockau W., Kahmann U., Kopertekh L., Staiger D., Pistorius E.K., Reuther J. (2009). Tuber-specific cphA expression to enhance cyanophycin production in potatoes. Plant. Biotechnol. J..

[19] Castanon S., Martin-Alonso J.M., Marin M.S., Boga J.A., Alonso P., Parra F., Ordas R.J. (2002). The effect of the promoter on expression of VP60 gene from rabbit hemorrhagic disease virus in potato plants. Plant. Sci..

[20] Garbarino J.E., Oosumi T., Belknap W.R. (1995). Isolation of a polyubiquitin promoter and its expression in transgenic potato plants. Plant. Physiol..

[21] McCue K.F., Ponciano G., Rockhold D.R., Whitworth J.L., Gray S.M., Fofanov Y., Belknap W.R. (2012). Generation of PVY Coat Protein siRNAs in Transgenic Potatoes Resistant to PVY. Am. J. Potato Res..

[22] Duan H., Richael C., Rommens C.M. (2012). Overexpression of the wild potato *eIF4E 1* variant *Eva1* elicits *Potato virus Y* resistance in plants silenced for native *eIF4E-1*. Transgenic Res..

[23] Neumann K., Stephan D.P., Ziegler K., Hühns M., Broer I., Lockau W., Pistorius E.K. (2005). Production of cyanophycin, a suitable source for the biodegradable polymer polyaspartate, in transgenic plants. Plant. Biotechnol. J..

[24] Meiyalaghan S., Takla M.F., Jaimess O., Yongjin S., Davidson M.M., Cooper P.A., Barrell P.J., Jacobs M.E., Wratten S.D., Conner A.J. (2005). Evaluation of transgenic approaches for controlling tuber moth in potatoes. Commun. Agric. Appl. Biol. Sci..

[25] Mohan S., Meiyalaghan S., Latimer J.M., Gatehouse M.L., Monaghan K.S., Vanga B.R., Pitman A.R., Jones E.E., Conner A.J., Jacobs J.M.E. (2014). GSL2 over-expression confers resistance to *Pectobacterium atrosepticum* in potato. Theor. Appl. Genet..

[26] Miroshnichenko D., Timerbaev V., Okuneva A., Klementyeva A., Sidorova T., Pushin A., Dolgov S. (2020). Enhancement of resistance to PVY in intragenic marker-free potato plants by RNAi-mediated silencing of eIF4E translation initiation factors. Plant. Cell Tissue Organ. Cult..

[27] Li M., Xie C., Song B., Ou Y., Lin Y., Liu X., Zhang H., Liu J. (2015). Construction of efficient, tuber-specific, and cold-inducible promoters in potato. Plant. Sci..

[28] Zhen W., Chen X., Liang H., Hu Y., Gao Y., Lin Z. (2000). Enhanced late blight resistance of transgenic potato expressing glucose oxidase under the control of pathogen-inducible promoter. Chin. Sci. Bull..

[29] Jefferson A., Kavanagh A., Bevan W. (1987). GUS fusion: Glucuronidase as a sensitive and versatile gene fusion marker in higher plants. EMBO J..

[30] Halterman D., Guenthner J., Collinge S., Butler N., Douches D. (2016). Biotech potatoes in the 21st century: 20 years since the first biotech potato. Am. J. Potato Res..

[31] Pfister B., Zeeman S.C. (2016). Formation of starch in plant cells. Cell Mol. Life Sci..

[32] Grierson C., Du J.S., de Torres Zabala M., Beggs K., Smith C., Holdsworth M., Bevan M. (1994). Separate cis sequences and trans factors direct metabolic and developmental regulation of a potato tuber storage protein gene. Plant. J..

[33] Zourelidou M., de Torres-Zabala M., Smith C., Bevan M.W. (2002). Storekeeper defines a new class of plant-specific DNA-binding proteins and is a putative regulator of patatin expression. Plant. J..

[34] Choi H.-i., Baek S.-Y., Kim S.Y. (2017). MYB class transcription factors bind to the tuber-specific and sucrose-response element of a class-I patatin promoter. Plant. Biotechnol. Rep..

[35] Jefferson R., Goldsbrough A., Bevan M. (1990). Transcriptional regulation of a patatin-1 gene in potato. Plant. Mol. Biol..

[36] Steege G., Nieboer M., Swaving J., Tempelaar M.J. (1992). Potato granule-bound starch synthase promoter-controlled GUS expression: Regulation of expression after transient and stable transformation. Plant. Mol. Biol..

[37] Kluth A., Sprunck S., Becker D., Lörz H., Lütticke S. (2002). 5′ deletion of a gbss1 promoter region from wheat leads to changes in tissue and developmental specificities. Plant. Mol. Biol..

[38] Visser R.G.F., Stolte A., Jacobsen E. (1991). Expression of a chimaeric granule-bound starch synthase-GUS gene in transgenic potato plants. Plant. Mol. Biol..

[39] Aminedi R., Das N. (2014). Class I patatin genes from potato (*Solanum tuberosum* L.) cultivars: Molecular cloning, sequence comparison, prediction of diverse cis-regulatory motifs, and assessment of the promoter activities under field and in vitro conditions. Vitr. Cell Dev. Biol. Plant.

[40] Heilersig B.H.J.B., Loonen A.E.H.M., Janssen E.M., Wolters A.M.A., Visser R.G.F. (2006). Efficiency of transcriptional gene silencing of GBSSI in potato depends on the promoter region that is used in an inverted repeat. Mol. Genet. Genom..

[41] Nap J.P., van Spanje M., Dirkse W.G., Baarda G., Mlynárová L., Loonen A., Grondhuis P., Stiekema W.J. (1993). Activity of promoter of the Lhca3.St.1 gene, encoding the potato apoprotein 2 of the light-harvesting complex of photosystem I, in transgenic potato and tobacco plants. Plant. Mol. Biol..

[42] Annadana S., Udayakumar M., de Jong J., Nap J.P. (2001). The potato *Lhca*3.St.1 promoter confers high and stable transgene expression in chrysanthemum, in contrast to CaMV-based promoters. Mol. Breed..

[43] Smirnova O.G., Tishchenko E.N., Ermakov A.A., Shumny V.K., Kanayama Y., Kochetov A. (2015). Promoters for transgenic horticultural plants. Abiotic Stress Biology in Horticultural Plants.

