# Supplementary material for: Evaluation of Plant-Derived Promoters for Constitutive and Tissue-Specific Gene Expression in Potato
Source: Plants (Basel). 2020 Nov 9;9(11):1520. doi: 10.3390/plants9111520 (PMC7696379; doi:10.3390/plants9111520)
Supplement: Supplementary file 1 [file plants-09-01520-s001.pdf]

Supplementary Table S1. Primers used for molecular analysis of transgenic potato plants

Primer name	Sequence (5′ – 3′)	Amplicon size (bp)	Destination
prL3-F	ACTGAGCTCGGCGCGCCTGGAAACGTTTAGTAAAATAGCC	995	Cloning the promoter of StLhca3
prL3-R	ACTGGTACCAATTTTTCTTCTCTTTTTTTTTGTTTTTG		
prUbi-F	ACTGAGCTCGGCGCGCCGGAATCTAATACTTACCTCTTAG	1222	Cloning the promoter of StUBi
prUbi-R	ACTGGTACCCTGCAAATTCATAAAAACAACAATC		
GBSSfor	GCAAGCTTTAACGAGATAGAAAATTATGTTACT	1003	Cloning the promoter of StGBSS
GBSSrev	TGTCTAGATGCATGAAATCAGAAATAATTGGAG		
B33for	CCCAAGCTTATGTTGCCATATAGAGTAG	1757	Cloning the promoter of StPat
B33rev	TCGGGGATCCTTTGCAAATGTTCAAAGTG		
35S-F	CTGCCGACAGTGGTCCCAAAGATGGACCC	1151	PCR analysis for 35S-gusA fusion
GUS2-R	GAATCCTTTGCCACGCAAGTCCGCATCTT		
prL3(538)-F	GTTATCATTATACCGTTAGAAGC	777	PCR analysis for Lhca3-gusA fusion
Gus-R	TCTGCATCGGCGAACTGATCGTTA		
intUbi-F	CAATTGGAGTTTTCCCCGTTGTTTTG	609	PCR analysis for ubi-gusA fusion
Gus-R	TCTGCATCGGCGAACTGATCGTTA		
GBg-F	TACTAGGAGACAGAACCGGACGGCC	488	PCR analysis for GBSS-gusA fusion
Gus-R	TCTGCATCGGCGAACTGATCGTTA		
B33g-F	CCCTCAAGAAGGACATTTGCGGTG	496	PCR analysis for Pat-gusA fusion
Gus-R	TCTGCATCGGCGAACTGATCGTTA		
nptII-1	GCTATGACTGGGCACAACAGACAATC	381	PCR analysis for nptII gene insertion
nptII-2	TCCGAGTACGTGCTCGCTCGA		
